# Three-Dimensional Microscopic Characteristics of the Human Uterine Cervix Evaluated by Microtomography

**DOI:** 10.3390/diagnostics15050603

**Published:** 2025-03-02

**Authors:** Ana Paula Pinho Matos, Osvaldo Luiz Aranda, Edson Marchiori, Alessandra Silveira Machado, Adriana José Da Penha Moreira, Heron Werner, Edward Araujo Júnior, Roberta Granese, Gloria Calagna, Pedro Teixeira Castro

**Affiliations:** 1 Department of Radiology, Federal University of Rio de Janeiro (UFRJ), Rio de Janeiro 21941-617, RJ, Brazil; anapaulapmatos@gmail.com (A.P.P.M.); edmarchiori@gmail.com (E.M.); heronwerner@hotmail.com (H.W.); pedrotcastro@gmail.com (P.T.C.); 2 Department of Obstetrics and Gynecology, Vassouras University, Vassouras 27700-000, RJ, Brazil; osvaldoaranda@uol.com.br; 3 Nuclear Engineering Program (PEN/COPPE), Federal University of Rio de Janeiro (UFRJ), Rio de Janeiro 21941-914, RJ, Brazil; alemachado@lin.ufrj.br (A.S.M.); adrianajpmoreira@coppe.ufrj.br (A.J.D.P.M.); 4 Department of Obstetrics, Paulista School of Medicine, Federal University of São Paulo (EPM-UNIFESP), São Paulo 04023-062, SP, Brazil; araujojred@terra.com.br; 5 Discipline of Woman Health, Municipal University of São Caetano do Sul (USCS), São Caetano do Sul 09521-160, SP, Brazil; 6 Department of Biomedical and Dental Sciences and Morphofunctional Imaging, “G. Martino” University Hospital, 98100 Messina, Italy; robertagr74@gmail.com; 7 Villa Sofia Cervello Hospital, University of Palermo, 90100 Palermo, Italy

**Keywords:** uterine cervix, microtomography, microscopy, two-dimensional image, three-dimensional image

## Abstract

**Objectives:** To analyze the microscopic anatomy of the human uterine cervix in two-dimensional (2D) and three-dimensional (3D) images obtained by microtomography (microCT). **Methods:** Human uterine cervixes surgically removed for benign gynecologic conditions were immersed in formalin and iodine solution for more than 72 h and images were acquired by microtomography. **Results:** In total, 10 cervical specimens were evaluated. The images provided by microCT allowed the study of the vaginal squamous epithelium, demonstrated microscopic 3D images of the metaplastic process between the exo and endocervix, and demonstrated the effects of metaplastic transformation on the thickness of the endocervical epithelium. Also reconstructed in 3D the endocervical folds and the repercussions of the metaplastic process on the endocervix, the changes of the endocervical epithelium along the cervical lumen and the relationship between the endocervix epithelium from the internal os and endometrium. In addition, 2D images could demonstrate the difference in tissue orientation of the collagen on the cervical stroma in a large field of view. **Conclusions:** MicroCT could demonstrate the microscopic anatomy of the human uterine cervix in 2D and 3D images, including the different stages of metaplastic process of the endocervical epithelium and reconstructed the endocervical lumen in 3D, preserving its natural anatomy without any mechanical effect for its dilatation.

## 1. Introduction

Despite its seemingly simple anatomy, the uterine cervix (UC) has complex functions throughout a woman’s life. From the isolation of the uterine cavity from vaginal microorganisms beginning in the prepubertal period, to sexual functions that can be extended after menopause, the UC is an organ that connects two different environments. To fulfill its functions, the UC changes according to the age of the woman, the cyclical hormones, and the presence of an implanted ovum [1].

The anatomical study of this simple, multifunctional organ has been documented since the second century when it was called the neck of the uterus by Soranus [2]. Over the centuries, the anatomy of the UC has been iconographically represented by drawings, as well as macroscopic, and microscopic studies. In the 20th century, three-dimensional (3D) medical imaging provided a spatial perspective of the anatomy of the female genital tract, from in vivo and ex vivo specimens [3,4]. However, artifacts and low-resolution images reduce the possibility of adequate perceiving the spatial relationship of different tissues on the cervix [3].

Cervical cancer is the fourth most common cancer among women worldwide [5], and 85% of cervical cancers occur in women living in low– to middle-income countries [6]. The origin of most cervical cancers occurs during the transformation of intracervical epithelium in extracervical epithelium, on the squamous-columnar junction [7]. Recently, human ex vivo specimens have been studied using microtomography (microCT), which allows the visualization of human organs in 3D and improves the perception of 3D relationships between tissues at the microscopic scale [8]. The purpose of this study is to analyze the three-dimensional anatomy of the human UC at a microscopic scale, the characteristics of the external and internal cervical epithelium, and the effects of metaplastic transformation on both the epithelium and squamocolumnar junction. Additionally, this study aims to evaluate the tridimensional characteristics of the cervical stroma, and its relation to internal and external os, analyzed by two-dimensional (2D) and 3D images provided by microCT.

## 2. Methods

### 2.1. Specimens

This analysis is part of a study using microCT to image human tissues and organs, approved by the Ethics Committee of University of Vassouras (CAAE 56031916.0.0000.5290). A prospective case selection was performed and patients undergoing hysterectomy for benign gynecological conditions (uterine fibroids, adenomyosis, and abnormal uterine bleeding) were included in this study. Patients were invited to participate in this study and signed an informed consent form.

Specimens were fixed in 10% formalin solution for more than 24 h at room temperature to ensure proper preservation of cellular and tissue architecture [9]. The specimens were washed twice with water and, for contrast enhancement between different soft tissues, the specimens were immersed in a 10% formalin and 10% Lugol solution diluted in physiological solution for more than 72 h. The specimens were washed of staining solution to reduce excessive saturation on the surface. The specimens were fixed on Styrofoam for support during image acquisition to reduce artifacts. In the course of the imaging capture, the specimen was rotated on its biggest axis, thus allowing capture in different angles. After image acquisition, the specimens were immersed in 10% formalin solution to remove the iodine concentration and were submitted to traditional histologic studies.

### 2.2. MicroCT

The General Electric Phoenix VTomeX (General Electric; Wunstorf, Germany) was used for imaging acquisition. The parameters for acquisition, including energy, current, and exposure time, were adapted for each specimen for better contrast between the cervical soft tissues.

### 2.3. MicroCT Parameters

The image acquisition parameters were energy between 50 and 130 kV (median 98 kV), exposure time between 220 and 480 ms (median 333 ms), current between 220 and 480 μA (median 307 μA), scan duration between 44 and 72 min (median 56 min), and voxel size between 22 and 39 μm (median 32 μm) (Table 1).

After the capture of the images of the specimens, the software NRECON version 1.7.4.5 (Bruker; Kontich, Belgium) was used for gathering the 2D X-ray images in a volumetric image for 3D analysis. With the volumetric image formed, the multiplanar analysis of the 3D volumetric image demonstrating the 2D appearance of the specimens in all the 3 planes was performed using the DataViewer (version 1.5.6.2 64-bit 2017; Bruker; Kontich, Belgium). The analysis and 3D image reconstruction were performed using CTVox version 1.16 (Bruker; Kontich, Belgium).

## 3. Results

A total of 10 human uterine cervixes from 10 patients who agreed to participate in this study were analyzed. The age of the patients ranged from 39 to 48 years (median 45 years). Demographic information of the patients is presented in Table S1. In nine cases, the uterine cervixes were anatomically normal. In one case, however, mechanical compression and distortion of the cervix caused by multiple Naboth cysts were observed. Surgery was suggested due to a lack of clinical response to conservative treatment and compressive effects of the enlarged uterus due to fibroids. All cervixes were removed through abdominal incisions after total hysterectomy. The extrafascial technique was used in all cases. In all specimens, the uterine cervix presented the vaginal segment (ectocervix), the entire abdominal part of the cervixes, including internal os. Laterally, the cervixes were removed, preserving the parametrium to avoid intra- and post-operative complications. In some specimens, the uterine segment was also examined.

### 3.1. Contrast

For proper contrast between different soft tissues, the iodine solution must penetrate the tissue throughout its volume. In eight specimens, the contrast was able to adequately penetrate the UC. In two specimens, the contrast was unable to differentiate the medial segment of the subepithelial stroma. 

### 3.2. Evaluation of Specimen Images

Sufficient impregnation of contrast in the specimens allowed the study of the microscopic anatomy of the UC in 2D and 3D images. The 2D images could be viewed in any selected plane and in any direction. Using the DataViewer software (version 1.5.6.2 64-bit 2017; Bruker; Kontich, Belgium), 2D images of specimens could be virtually sliced in any direction with simultaneous visualization of the specimen in different planes. The section of interest could also be visualized in all three planes simultaneously (Figure S1). The contrast of the images could be adjusted to better visualize the region of interest for the observer. The 3D images were generated using the CTVox software version 1.16 (Bruker; Kontich, Belgium).

### 3.3. The Anatomy of the Uterine Cervix

**Figure 1:**
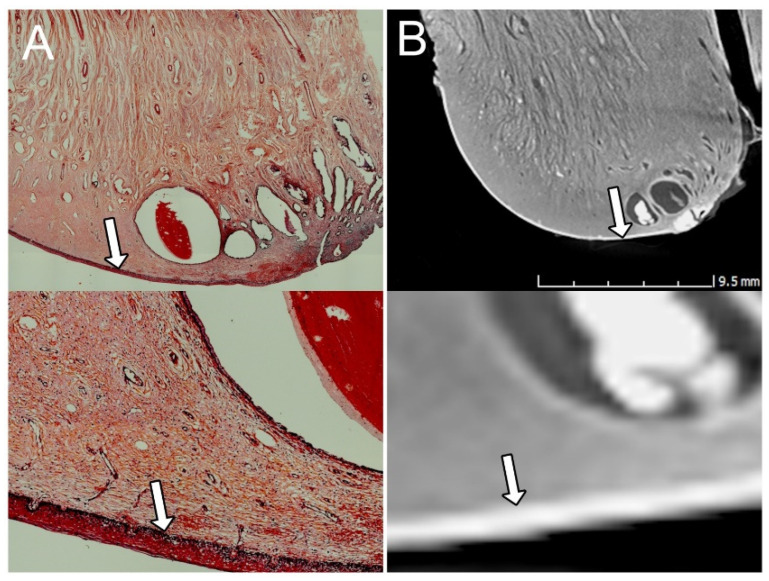
The vaginal mucosa covering the uterine cervix. The vaginal mucosa and its stratum corneum are visible (arrow) in the conventional microscopic image (**A**) and in the X-ray microtomographic image (**B**). The ability of this epithelium to absorb iodine is demonstrated by the intense contrast caption, shown as a prominent white appearance (radiopaque) in the radiographic image (**B**). Also, note the difference of the imaging definition at the cellular level between the two techniques (lower images): despite the capacity of acquisition of images at a microscopic scale, the detailed morphology at cellular scale could not be achieved by microCT.

The UC is the lowest part of the uterus and has the histologic characteristics of a transition from a fibromuscular structure in the cranial part adjacent to the uterine segment to a fibrous structure in the portio vaginalis. The UC is divided into two parts: the supravaginal part, lying above the vagina in the abdominal segment, and the vaginal part protruding into the vagina [10]. The stratum corneum can be seen in 2D microCT images as the external part of the endocervix. This feature also correlates with the traditional microscopic image in 2D (Figure 1).

Similar to in vivo specimens, iodine was more intensely present in this part of the cervix, more radiopaque in the microCT X-ray image. However, the limitation of microCT becomes apparent when it is noticed that the scale of the image pixels generated by microCT cannot show the images in the same scale as the traditional optical microscopy. In addition, the X-ray images produce images according to the X-ray opacity of the tissue and its ability to absorb contrast.

### 3.4. Transformation of the Endocervical Epithelium—Metaplastic Transformation

**Figure 2:**
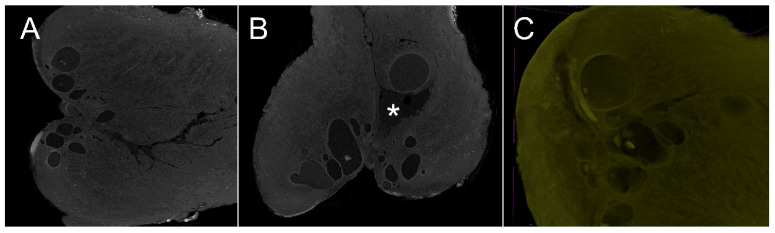
(**A**,**B**) Coronal and sagittal 2D images of the uterine cervix. The multiple cystic images correspond to Naboth cysts. In (**B**), the intracervical Naboth cyst distorts the cervical lumen with significant discontinuity between the cervical epithelium (asterisk). (**C**) shows 3D reconstruction of the same uterine cervix and the spatial relationship between the Naboth cysts and the cervical epithelium.

Metaplasia is the transformation of cells or tissues into other cells or tissues. Transformation of mature endocervical epithelium into mature squamous epithelial cells is a physiological process of the UC when columnar epithelium is exposed to the vaginal environment. After the transformation of the columnar epithelium, the metaplastic areas appear as normal ectocervical squamous epithelium. This transformation can be seen by sectioning the UC when the residual crypts form the Naboth cysts. The Naboth cysts were easily visualized in 2D and 3D images obtained by microCT. In one specimen, the presence of multiple Naboth cysts distorted the anatomy of the cervical canal. The cyst pushed the endocervical epithelium caudally, creating an irregular cavity in the cervical lumen (Figure 2).

**Figure 3:**
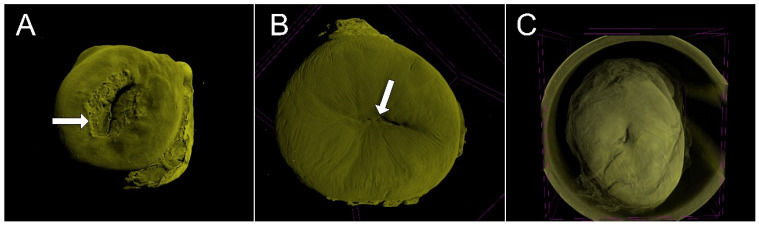
The “virtual colposcopy” of uterine cervix specimens generated by microCT images: the squamocolumnar junctions and their visibility at colposcopy. In (**A**), the squamocolumnar junction (arrow) is completely visible (type 1); in (**B**), the squamocolumnar junction (arrow) is partially visible (type 2); and in (**C**), the squamocolumnar junction is not visible (type 3).

**Figure 4:**
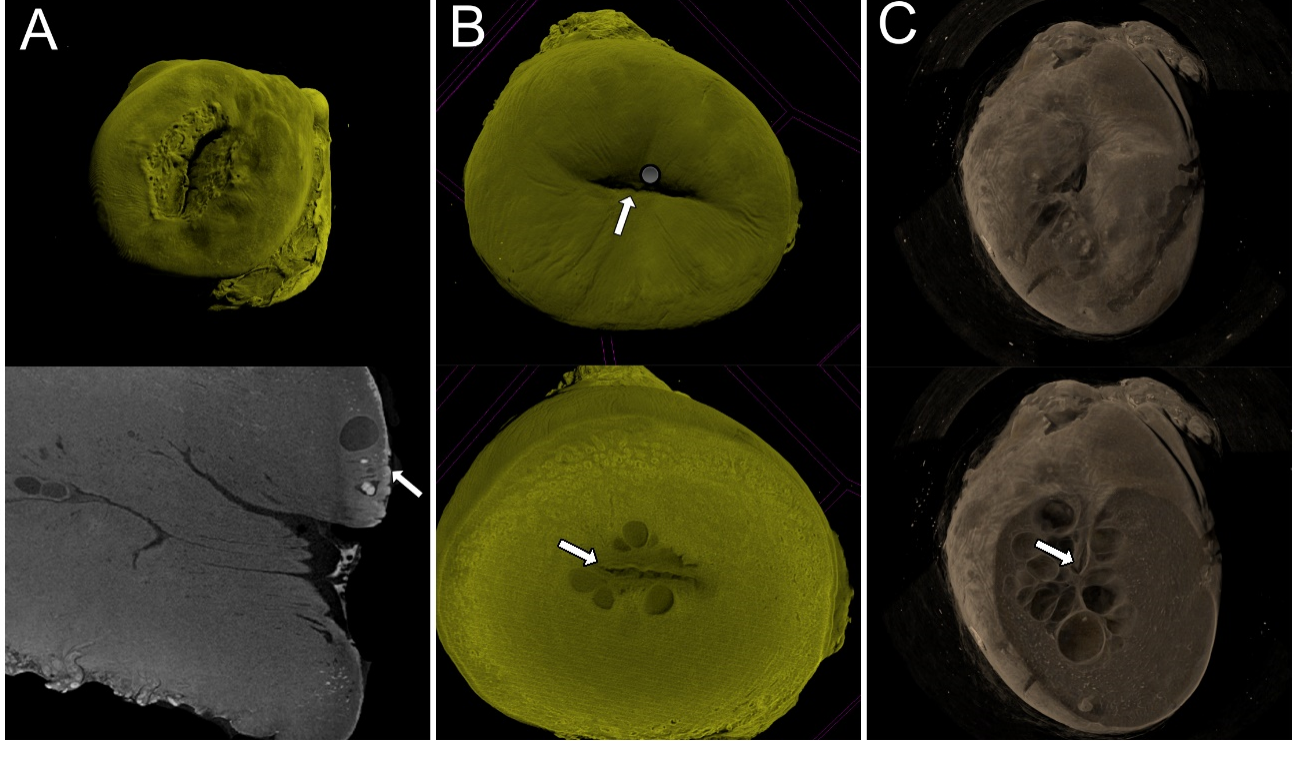
The uterine cervix and the squamocolumnar junction from different perspectives. In (**A**), the fully visible squamocolumnar junction is shown in 3D and below in 2D images, demonstrating the area of metaplastic transformation (arrow). (**B**) shows the partially visible squamocolumnar junction (arrow) after virtual cervical sectioning. In (**C**), the squamocolumnar junction not visible in “virtual colposcopy” can be seen in a virtual section of the cervix, demonstrating the most caudal epithelium surrounded by Naboth cysts.

The metaplastic process was well demonstrated in all ten specimens. The transformation zone could be visualized in 2D and 3D images, and the border between the squamous and glandular epithelium was clearly demonstrated. The squamocolumnar junction was well visualized, especially in the 3D images. Small round openings on the surface of the squamous epithelium demonstrated the opening of the endocervical epithelium under the epithelium under metaplasia. The specimen presented the main features of UC at colposcopy, with the squamocolumnar junction completely visible (type 1), partially visible (type 2), and not visible (type 3) (Figure 3). The 3D images of microCT allowed the sectioning of the UC in all planes, allowing the visualization of the squamocolumnar junction in 2D or 3D images (Figure 4).

### 3.5. The Endocervical Anatomy

**Figure 5:**
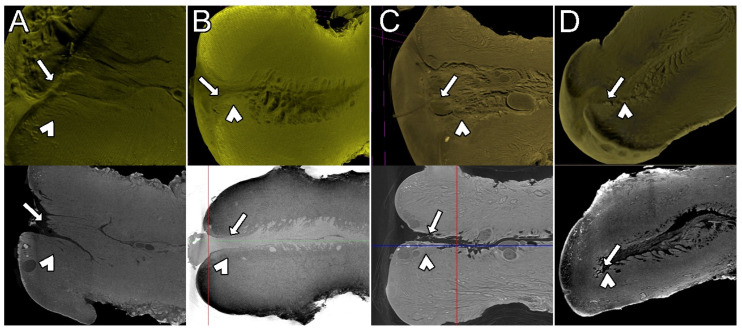
The endocervical epithelium and its relationship to the external os and cervical stroma. (**A**) shows the epithelial folds toward the vaginal cavity (arrowhead), presenting their extremity as a “bunch of grapes”. The epithelium is forced in a parallel direction, with its most external extremity of “small polypoid epithelium”, creating the appearance of a “bunch of grapes” visualized in colposcopic examinations (arrow). In (**B**), the epithelial folds still have a thick part of the external os (arrowhead). The pattern of parallel folds can be seen as the metaplasia progresses. However, there is a significant reduction in the number of endothelial folds and their thickness, with a more relevant sub-epithelial stroma. The shape of the crypts is similar to the *plicae palmatae* described in classic textbooks. In (**C**), the epithelium becomes scarce and simultaneously the cervical stroma occupies the external os of the uterine cervix covered by the squamous epithelium. (**D**) shows the metaplastic phenomena, the absence of visible cervical endocervical epithelium on colposcopic examination. The *portio vaginalis* is completely covered by metaplastic squamous epithelium. The columnar epithelium has lost its thickness and few folds, with a cobblestone appearance visible in 2D and 3D images.

The endocervical epithelium showed different characteristics in the specimens, mostly influenced by the metaplastic transformation. MicroCT images allowed the 3D visualization of the different types of squamocolumnar junctions and the corresponding 3D anatomy of the endocervical epithelium. The epithelium presenting outside the external os has a “grape” appearance. This appearance is created by the compressed folds of the epithelium, forcing the epithelium in a parallel direction with its outermost extremity of polypoid epithelium, creating the “grape” appearance visualized in colposcopic examinations (arrow, Figure 5A). The epithelial folds represent a thick portion of the external os (arrowhead, Figure 5A). This interesting pattern of parallel folds can be seen as metaplasia progresses. However, there is a significant reduction in the number of epithelial folds and their thickness (Figure 5B). The epithelium becomes scarce, and the stroma occupies the external os of the UC (Figure 5C). In the final stage of metaplasia, there is no visible cervical endothelium on colposcopic examination. The *portio vaginalis* is completely covered by metaplastic squamous epithelium. The columnar epithelium lost its thickness and some folds with a cobblestone appearance were visible in 2D and 3D images (Figure 5D).

### 3.6. The Endocervical Canal Beyond the External os

**Figure 6:**
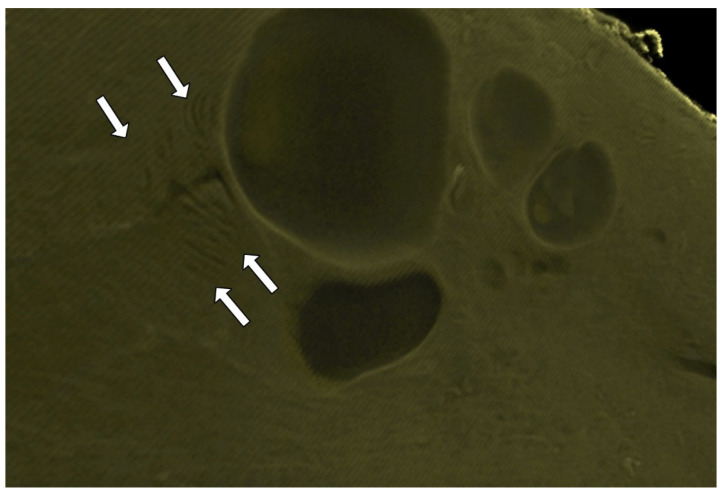
The cervical epithelial folds (arrows) are preserved despite the presence of larger cysts in this specimen.

The specimens showed different patterns of endocervical canal folding and consequently different sizes and complexities in the lumen. The natural history of trauma secondary to labor and natural epithelial obstruction with cystic formations can be demonstrated by the deformation of the luminal space in some specimens (Video S1). In other specimens, however, the presence of cysts in the cervical epithelium, even larger cysts, had little effect on the cervical lumen and, most importantly, no apparent change in the cervical folds (Figure 6, Video S2).

The cervical mucus could be observed in some specimens. The thick and dense mucus filled the glands and remained inside the cervix even after the period of immersion in formalin and iodine solution. The mucus did not absorb iodine due to its characteristic water concentration (95–99%). The presence of mucus created an interface between the lumen and the epithelium, facilitating the 3D visualization of the structural appearance of the cervical epithelium from the luminal perspective, without the necessity of mechanical dilatation. The epithelium presents crypts on 2D images, following a regular pattern of size and spacing between epithelial layers. The crypts tend to be perpendicular to the lumen or have their layers facing the external os. The size of the epithelial crypts is continuous, decreasing in size with proximity to the internal os.

**Figure 7:**
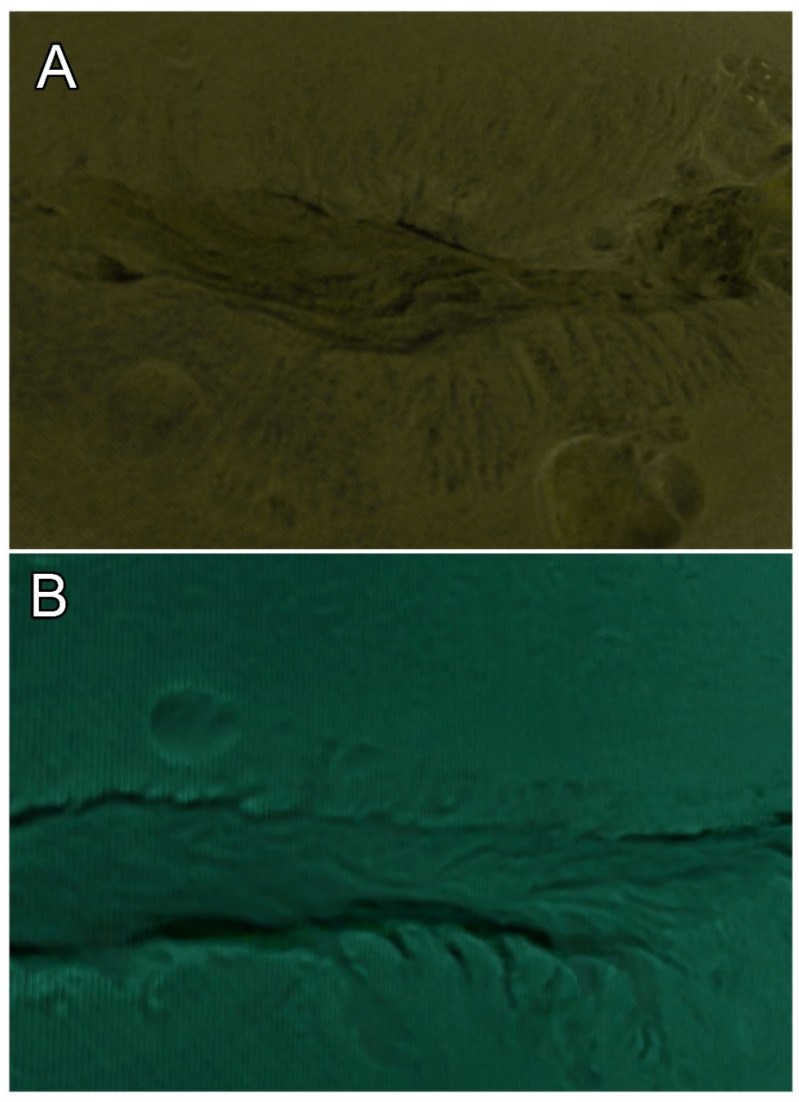
In (**A**,**B**); epithelial folds and crypts. The section of the 3D image of the endocervix shows the direction of the crypts, which are directed towards the external os. Their outer border can be seen in the direction of the center of the lumen and with the appearance of fusion in waves of folds.

The 3D images of the epithelial lining inside the endocervix show an unpredictable and complex pattern. The gathering of the epithelial lining creates folds that have their greatest length parallel to the cervical axis. The folds have their axis toward the exocervix (Figure 7) and are reduced in size near the internal os, where parallel folds can be seen, and become sparse and thin in advanced metaplastic cervixes.

**Figure 8:**
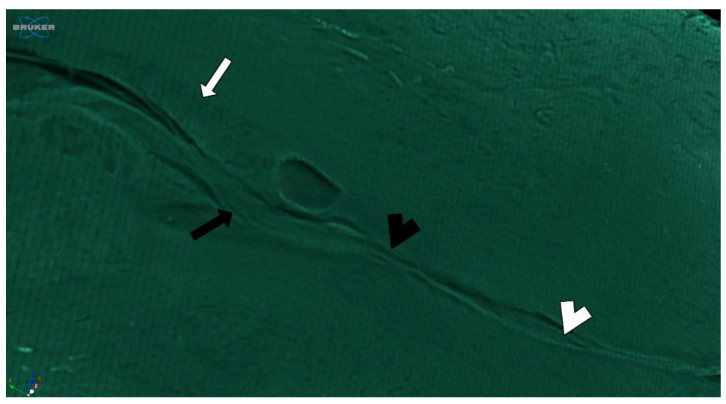
The transition from the endocervix to the endometrium. The folds of the endocervical epithelium became sparse, predictable, and organized (arrow) to a thin, simple, and rudimentary endometrial tissue (arrowhead). The subtle change between tissues has no other anatomical marker on the uterine cervix.

The internal os is characterized by a subtle transition between uterine tissues, with a complex endocervical epithelium becoming more organized and predictable to a thin endometrial gland that is smaller in size compared to the endocervical epithelium (Figure 8). No other differences were observed in the subepithelial stroma. The Video S3 shows the main anatomical features of the UC.

**Figure 9:**
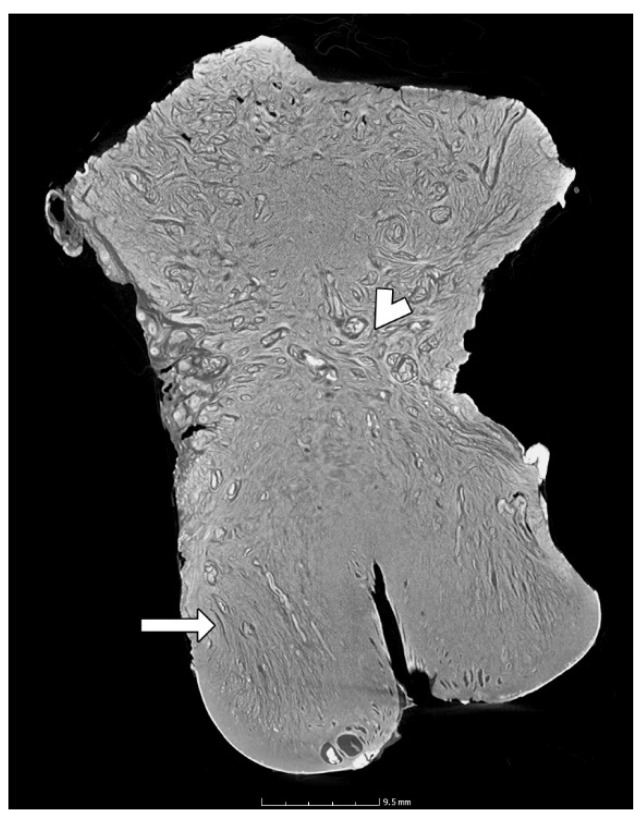
Visualization of the stroma. The cervical fibers appear to be parallel to the luminal axis (arrow), but there is a change in direction in the internal os (arrowhead).

Using 2D images, the cervical stroma shows an alteration in the orientation of the directions of the collagen fibers. The stromal fibers appear to be parallel to the endocervical lumen along their entire length. However, in the internal os, there is an apparent change to a perpendicular direction (Figure 9).

## 4. Discussion

This case series demonstrates aspects of human UC anatomy at the microscopic level, in 2D and 3D images provided by microCT. Throughout the last century, other X-ray technologies have imaged the cervix in vivo, but the improvement of soft tissue contrast in ex vivo biological specimens has allowed the acquisition of cervical images with high resolution [11]. MicroCT is a non-destructive technology, allowing the acquisition of images without destroying specimens for further histologic study [12,13].

**Figure 10:**
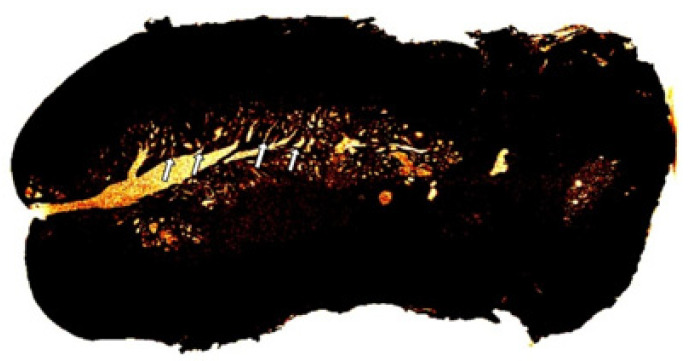
The crypts open into the cervical lumen. The arrows indicate the opening of the crypts, and the 2D image shows the large number of gland-like structures that will be opened in the lumen.

The endocervix produces mucus at rate of 20–60 mg/day in reproductive age, and this production increases 10-fold in the preovulatory phase of the cycle, reaching 700 mg/day [14]. Traditional studies consider the presence of 100 “mucus-secreting glandular-like units” in the cervical canal [15]. In this series, the 3D images allowed the visualization of the crypts with details due to the presence of mucus. Also, the traditional studies for estimating the number of crypts were based on 2D histologic slides, which made the estimation of crypts inaccurate. Figure 10 shows the spatial distribution of glands in a 2D image.

The connective tissue of the cervical stroma is divided into two segments: the superficial zone and the subepithelial stroma, which is rich in interstitial fluid and may be infiltrated by adjacent inflammatory exudates. The superficial zone is deeper and has a dense collagenous layer [16,17,18].

The possibility of visualizing the endocervical epithelium during colposcopic examination with optical instruments such as hysteroscopy is well known, but these instruments require additional distance to the inner part of the epithelium [19,20]. Creating a true distance for visualization requires the use of mechanical force, such as forceps, or increasing the intraluminal pressure with saline solution or gas, which compresses and distorts the structures. In this series, the presence of mucus in the microtomographic image of the cervix allowed the visualization of the 3D structure of the folds and crypts of the cervical epithelium without any additional mechanical effect.

MicroCT has been used for the study of human specimens since the early 1980s, but the development of methods to enhance contrast between different soft tissues of biological specimens allowed microCT to be used for the study of human soft tissues. From brain tissue [21] to fetal heart morphology [22], microCT has gained increasing interest in the three-dimensional study of microscopic anatomy. The addition of iodine solution as a soft tissue contrast agent has facilitated the specimen preparation process [23]. In human gynecological specimens, the 3D microscopic anatomy of ectopic pregnancy [24] and tubal changes [25] has been described using microCT.

The normal characteristics of the microanatomy of the fallopian tube were described, with detailed anatomy of the tubal mucosa [12]. The high definition of the tubal mucosa allowed segmentation of the tubal mucosa and creation of a volumetric image of the tubal lumen, allowing virtual navigation within the normal fallopian tube [26]. The high definition of the endocervical lumen was also observed in this series case. The microtomographic images allowed visualization of the folds created by the epithelium without external effects for luminal dilatation.

A study evaluating the anatomy of uterus and cervix of rats demonstrated the accuracy of microtomographic images when compared to classic histology methods. The traditional microscopy can achieve cellular and sub cellular at high-resolution when compared to microCT. However, in rats, microCT was very effective in the study of muscular fibers of the uterus [27]. In humans, the orientation of the cervical collagen–cellular fibers has been described based on 2D images for centuries, and recent studies have demonstrated the circumferential orientation of the uterine components in the internal os [28,29].

## 5. Conclusions

In summary, this study demonstrated the main anatomical features of the human UC from ex vivo specimen images provided by microCT, with special attention to the exhibition of the cervical lumen and its characteristics according to the stage of cervical metaplasia. In addition, the large field of view of microCT images allows visualization of the cervical stroma in 3D images. We believe that 3D microCT images may be an important tool for studying the orientation of cervical stroma fibers in human specimens, as recently described in animal studies [23].

## Figures and Tables

**Table 1:** Results of acquisition data of microCT images.

Acquisition Data	Voltage (kV)	Current (µA)	Exposure Time (ms)	Voxel Size (µm)	Number of Images	Number of Frames	Scan Time (Minute)
Result	98	307	333	32	1425	5	56

## Data Availability

The data presented in this study are available on request from the corresponding author.

## Supplementary Materials

The following supporting information can be downloaded at: https://www.mdpi.com/article/10.3390/diagnostics15050603/s1, Video S1. Uterine cervix with multiple Naboth cysts distorting the cervical lumen. Video S2. In some specimens, the presence of large cysts had little effect on the cervical lumen, with little effect on the folding arrangement of the epithelial endocervix. Video S3. 3D reconstruction of three different specimens. Figure S1. The visualization of the specimen in 2D views and the relation of the views in 3D by DataViewer.
