# Supplementary figures and images for: Three-Dimensional Microscopic Characteristics of the Human Uterine Cervix Evaluated by Microtomography
Source: Diagnostics (Basel). 2025 Mar 2;15(5):603. doi: 10.3390/diagnostics15050603 (PMC11898730; doi:10.3390/diagnostics15050603)

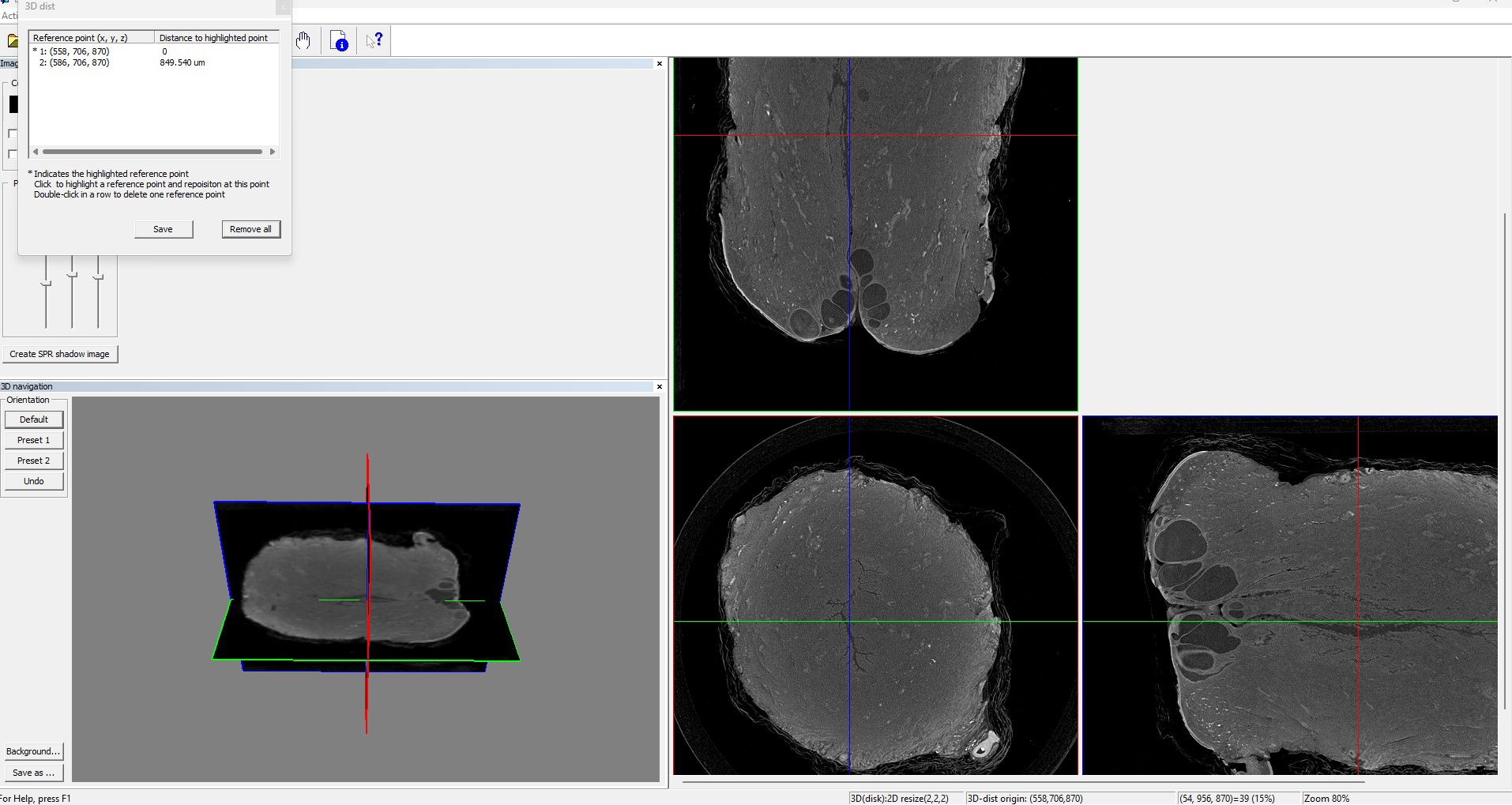

Supplement: Supplementary file 1 [file diagnostics-15-00603-s001.zip › Figure S1.jpg]
